# Dysfunctional Parvalbumin Neurons in Schizophrenia and the Pathway to the Clinical Application of Kv3 Channel Modulators

**DOI:** 10.3390/ijms25168696

**Published:** 2024-08-09

**Authors:** Masaya Yanagi, Mamoru Hashimoto

**Affiliations:** Department of Neuropsychiatry, Faculty of Medicine, Kindai University, 377-2 Ohnohigashi, Osaka-Sayama, Osaka 589-8511, Japan

**Keywords:** Kv3, parvalbumin, GABA, schizophrenia, targeted therapeutics

## Abstract

Based on the pathophysiological changes observed in schizophrenia, the gamma-aminobutyric acid (GABA) hypothesis may facilitate the development of targeted treatments for this disease. This hypothesis, mainly derived from postmortem brain results, postulates dysfunctions in a subset of GABAergic neurons, particularly parvalbumin-containing interneurons. In the cerebral cortex, the fast spike firing of parvalbumin-positive GABAergic interneurons is regulated by the Kv3.1 and Kv3.2 channels, which belong to a potassium channel subfamily. Decreased Kv3.1 levels have been observed in the prefrontal cortex of patients with schizophrenia, prompting the investigation of Kv3 channel modulators for the treatment of schizophrenia. However, biomarkers that capture the dysfunction of parvalbumin neurons are required for these modulators to be effective in the pharmacotherapy of schizophrenia. Electroencephalography and magnetoencephalography studies have demonstrated impairments in evoked gamma oscillations in patients with schizophrenia, which may reflect the dysfunction of cortical parvalbumin neurons. This review summarizes these topics and provides an overview of how the development of therapeutics that incorporate biomarkers could innovate the treatment of schizophrenia and potentially change the targets of pharmacotherapy.

## 1. Introduction

Schizophrenia is a psychiatric disorder that poses a significant social burden. It ranks high in the years lived with disability and also in the disability-adjusted life years, which is a comprehensive measure considering years lost due to illness, disability, and premature death [1]. Schizophrenia has a relatively high lifetime prevalence, which is estimated to be approximately 0.7% to 1% [2]. As this disease typically occurs from adolescence to early adulthood, it can cause a long-term decline in social function, leading to substantial economic and social burdens.

Schizophrenia is associated with positive (e.g., psychosis involving hallucinations and delusions) and negative (avolition, alogia, and affective flattening) symptoms, as well as cognitive impairment [2]. Antipsychotics have a certain effect on positive symptoms but negligible effects on negative symptoms and cognitive impairment. The stages of schizophrenia are prodromal, acute, and residual. Antipsychotics are highly effective for the first episode of psychosis in schizophrenia, i.e., the initial acute phase. It is estimated that over 80% of first-episode cases of schizophrenia achieve remission of positive symptoms [2]. However, due to the high risk of relapse, the proportion of patients who can maintain remission is significantly low. A 7-year follow-up study reported that only one-third of patients achieved social/vocational recovery [3]. In patients with schizophrenia who experience recurrent relapses, the dosage of antipsychotic medications is increased, leading to an increased risk of side effects.

At present, numerous antipsychotics are available, whereas all of their primary pharmacological mechanisms of action are principally on the dopamine D2 receptor [4]. Considering the insufficient effects of current medications for schizophrenia, there is clearly a need to develop a novel class of drugs with different mechanisms of action for this disease beyond the action on the D2 receptor. Based on these backgrounds, the development of first-in-class therapeutics that do not act on D2 receptors has been attempted, some to phase 3 of clinical trials. These include agents targeting glycine transporters and the subtypes of glutamate, serotonin, and acetylcholine receptors. However, to date, none of these agents have been approved for market use. Clinically available GABAergic agents, such as benzodiazepines, have rapid action for treating anxiety and insomnia and are also used adjunctively in the treatment of schizophrenia. The development of drugs focusing on the subtypes of GABAergic neurons may have therapeutic benefits for these symptoms, particularly in the context of schizophrenia. In light of these challenges, this review focuses on the perspective of gamma-aminobutyric acid (GABA) neurons, one of the representative pathophysiological hypotheses of this complex disorder, and provides an overview of recent trends in the development of novel drugs targeting the GABAergic system. In addition, it discusses the considerations for their clinical implementation.

## 2. Representative Pathophysiological Hypothesis in Schizophrenia

### 2.1. Dopamine Hypothesis: The Dawn of Pharmacotherapy

The pharmacotherapy of schizophrenia originated from the unexpected discovery of chlorpromazine as an effective treatment for schizophrenia in the 1950s [2,5]. Since then, the development of antipsychotics based on the pharmacological action of chlorpromazine has rapidly advanced, and many antipsychotics have been launched on the market. All of these antipsychotic drugs, whether typical or atypical, have an antagonistic effect on the D2 receptor. Even recently developed antipsychotics classified as D2 receptor partial agonists demonstrate functional activity that is close to antagonism [4]. The exception might be clozapine, which has been recognized as more effective than other antipsychotics for treatment-resistant schizophrenia and has limited D2 receptor antagonistic activity compared with other antipsychotics [4]. However, its mechanism of action remains unclear.

The successful development of antipsychotics based on D2 receptor antagonistic action for the treatment of schizophrenia led to the proposal of the dopamine hypothesis [6]. The dopamine hypothesis postulates that schizophrenia can be primarily caused by the abnormal function of dopamine. This hypothesis well represents the psychotic symptoms of schizophrenia. However, the limitation of this hypothesis is that it only explains the positive symptoms of the disease. Amphetamines, which promote dopamine release, L-dopa, increase dopamine transmission, and dopamine agonists can cause symptoms similar to the positive symptoms of schizophrenia, such as hallucinations and delusions if given repeatedly or at high doses [7]. The effects of antipsychotics are mainly on positive symptoms, with limited impact on negative symptoms and cognitive impairment. Furthermore, there is limited evidence of the dysfunction of dopaminergic neurons themselves in the pathophysiology of schizophrenia [7]. Based on these lines of evidence, it has been considered that further pathological mechanisms are involved in schizophrenia, even though the positive symptoms are attributed to dopaminergic dysregulation [6].

Despite recent vigorous biological research, the pathophysiology of the disease remains unknown. As schizophrenia is diagnosed as a syndrome based on clinical interviews, its biological pathophysiology is presumed to be heterogeneous. The complexity and heterogeneity of schizophrenia comprised several hypotheses for the pathophysiology of this disease, other than the dopamine hypothesis.

### 2.2. GABA Hypothesis: Disturbance of GABAergic Neurotransmission

The findings from postmortem brain studies of schizophrenia have shaped the GABA hypothesis in this disease [8]. GABA is the primary inhibitory neurotransmitter, and GABAergic neurons, which mainly release GABA, are widely distributed throughout the central nervous system. In the cerebral cortex, the majority of these neurons play a pivotal role as interneurons in the regulation of the activity levels of excitatory neurons, the principal neuronal type, glutamatergic neurons [9]. Studies that analyzed postmortem brain tissues from patients with schizophrenia have shown abnormalities in GABAergic neurotransmissions in the cerebral cortex. These include reduced expression of glutamic acid decarboxylase 67 (GAD67), a key enzyme for GABA synthesis, as well as abnormal expressions of GABA transporter 1 and GABA receptors in the cerebral cortex, mainly in the prefrontal lobe of patients with schizophrenia [8,10].

GABAergic neurons have a heterogeneous population and can be categorized into several subtypes distinguished by marker molecules, such as parvalbumin, somatostatin, and calretinin [8]. Parvalbumin and calretinin are calcium-binding proteins, whereas somatostatin is a neuropeptide; these molecules can be identified in brain tissues through immunohistochemistry. Among these subtypes, postmortem brain studies on patients with schizophrenia have reported several abnormalities in the parvalbumin neurons in the cerebral cortex. These include reduced levels of parvalbumin expression itself, reduced levels of GAD67 in parvalbumin neurons, and an increase in GABA-A receptors assumed to be compensatory to the reduced GABAergic neurotransmissions [8].

Parvalbumin-positive GABAergic interneurons (parvalbumin neurons) are thought to be involved in the control of higher-order brain functions requiring neural plasticity and learning [9]. The impairments in this cerebral GABAergic system have been implicated in cognitive impairments, such as impaired working memory, in patients with schizophrenia [8]. Based on the postmortem brain findings and their clinical implications, clinical trials investigated a GABA-A receptor agonist as a potential treatment for schizophrenia [11,12]. The initial trial showed promise, with improvements in neuropsychological tasks requiring working memory and a concomitant increase in induced gamma band power during the task in the electroencephalography (EEG) [11]. However, the subsequent trial of this drug for patients with schizophrenia failed to demonstrate a significant impact on overall cognitive function, including working memory, in patients with schizophrenia [12].

### 2.3. N-Methyl-D-Aspartate (NMDA) Hypothesis: A Comprehensive Hypothesis across the Entire Spectrum of Symptoms

The glutamate hypothesis postulates that abnormalities in glutamate, a primary excitatory neurotransmitter, cause schizophrenia. The NMDA hypothesis further specifies this notion, thereby emphasizing the critical role of NMDA receptor dysfunction. NMDA receptors are an ionotropic type of glutamate receptors that transmit excitatory signals to postsynaptic neurons when the glutamate released from the nerve terminals of excitatory neurons, called glutamatergic neurons, is received [13]. Administration of NMDA receptor antagonists, such as phencyclidine (PCP), has been reported to induce a range of symptoms in humans that resemble schizophrenia [13]. These symptoms include negative and positive symptoms as well as cognitive impairment.

The glutamate hypothesis of schizophrenia has been widely supported by several lines of biological evidence. This includes genetic studies demonstrating that genes involved in glutamate signaling are associated with schizophrenia [13] and neuroimaging studies employing proton magnetic resonance spectroscopy that have reported alterations of glutamine and glutamate acids in the prefrontal cortex and hippocampus of patients with schizophrenia [13]. Moreover, in animal model studies of schizophrenia involving mice that were acutely, subchronically, or chronically administered NMDA receptor antagonists, various types of behavioral abnormalities mimicking positive and negative symptoms, as well as the cognitive impairment of schizophrenia, have been identified [14].

Because the NMDA hypothesis provides a compelling explanation of the full range of symptoms in schizophrenia, several compounds have been explored as potential therapeutic agents for schizophrenia, including the glycine transporter 1 (GlyT1) inhibitor and mGlu2/3 receptor agonist, both of which are believed to modulate NMDA receptor function [15,16]. However, it is noteworthy that similar to the GABA hypothesis, drugs developed based on the NMDA hypothesis have not yet obtained market approval after failing to demonstrate efficacy in the clinical trials for patients with schizophrenia.

### 2.4. A Model Integrating the GABA and NMDA Hypotheses

The reductions in GABAergic neurotransmission and NMDA receptor hypofunction can lead to dopamine hyperactivity, which is implicated in the positive symptoms of schizophrenia [4]. Based on these individual neurotransmitter alterations that converge into dopamine hyperactivity, a comprehensive pathophysiological model of schizophrenia that incorporates both NMDA receptor abnormalities and dysfunction in GABAergic interneurons has emerged [4,17].

NMDA receptors are expressed in both glutamatergic and GABAergic neurons. Accumulating evidence from animal studies indicates that dysfunction of NMDA receptors expressed on parvalbumin interneurons mainly contributes to the pathophysiology of schizophrenia [17,18]. The parvalbumin neurons in the hippocampus and neocortex abundantly express NMDA receptors and receive more glutamatergic inputs than the pyramidal neurons in rodents [18]. Therefore, the NMDA receptor antagonists preferentially block NMDA receptors on these parvalbumin neurons [14,18]. Moreover, an animal model that selectively inhibited NMDA receptors, specifically on parvalbumin neurons, has demonstrated impairments in evoked gamma oscillations and working memory [19,20]. Also, the findings from the rodent study indicated that the NMDA receptors in these neurons could be the site of action of NMDA receptor antagonists, as these mice displayed blunted NMDA receptor antagonist-induced hyperlocomotor activity [19].

These findings in impaired neurotransmissions involving NMDA and GABA have led to the perception that the balance between excitatory and inhibitory neurotransmissions is essential in the pathophysiology of schizophrenia [21]. In recent years, there has been a growing interest in the use of an EEG as an indicator to evaluate the net balance between excitatory and inhibitory neural activity based on its noninvasive and relatively inexpensive utility as a tool to measure brain activity.

## 3. Utility of EEG for Targeted Therapeutics

### 3.1. EEG Findings in Schizophrenia

An EEG usually measures across a spectrum ranging from delta to gamma waves. In studies of resting-state EEGs, increased slow waves, such as delta and theta, have been reported in patients with schizophrenia [22]. Contrarily, during task-induced EEGs, a decrease in fast waves, particularly gamma oscillations, has been repeatedly reported [23]. Gamma oscillations are brain waves with a frequency of 30–100 Hz that can be detected through an EEG or magnetoencephalography (MEG). Cortical gamma oscillations are believed to be crucial for higher-order brain functions, such as perception, attention, and working memory [24]. Among the evidence in the task-induced EEG in patients with schizophrenia, there is high reproducibility in the findings, indicating that the auditory steady-state response (ASSR), using a periodic auditory stimuli rate of 40 Hz, is reduced in patients with schizophrenia [25,26]. The ASSR is the EEG (or MEG) response elicited by presenting periodic auditory tones at a specific frequency. ASSR potentials are largest in humans when elicited by periodic auditory stimuli at a frequency of approximately 40 Hz. Such a 40 Hz ASSR can be detected not only in humans but also in rodents, making it a promising translational biomarker that can connect human and animal models in schizophrenia.

### 3.2. Role of Parvalbumin Neurons in Gamma Oscillations

The coordinated interplay between parvalbumin neurons and glutamate neurons is crucial for the generation and maintenance of gamma oscillations [27]. Rodent studies have demonstrated that parvalbumin interneurons are important in the generation of gamma oscillations [9]. An optogenetic study in mice reported that the specific activation of cortical parvalbumin neurons using a 40 Hz stimulation induced gamma oscillations, whereas the same stimulation to glutamate neurons did not significantly increase gamma oscillations [28]. In humans, pharmacological validation has been conducted using the GABA-A receptor agonist, lorazepam, and the NMDA antagonist, memantine, in healthy subjects [29]. This study demonstrated a specific increase in the 40 Hz ASSR in the auditory cortex with lorazepam but not with memantine. These findings indicate that task-related gamma oscillations, particularly the 40 Hz ASSR, can be a biomarker for assessing the efficacy of drugs targeting parvalbumin neurons in patients with schizophrenia. However, further research is required to establish the critical role of parvalbumin neurons in the 40 Hz ASSR.

## 4. Kv3.1: A Potential Therapeutic Target of Parvalbumin Neurons

Parvalbumin neurons are also known as fast-spiking neurons owing to their characteristic high-frequency firing. In recent years, efforts have been made to develop drugs targeting the firing of parvalbumin neurons.

### 4.1. A Brief Overview of Kv3.1 and Kv3.2

Rodent studies have demonstrated that the parvalbumin neurons in the cerebral cortex express two types of potassium channels: Kv3.1 and Kv3.2 [30]. These potassium channels belong to the Kv3 family, which consists of four types of potassium channels: Kv3.1 to Kv3.4. Kv3 is a group of voltage-gated channels with a high activation threshold and rapid activation and inactivation kinetics, enabling the high-frequency firing of the neurons [30]. The high-frequency firing of parvalbumin neurons critically depends on the expression of Kv3.1 and Kv3.2 in the cerebral cortex. Studies that used knockout mice lacking Kv3.1 or Kv3.2 reported a reduction in the high-frequency firing of parvalbumin neurons [31,32]. In juvenile mice examined at around P13 of age, the parvalbumin neurons lack maturity for high-frequency firing [33]. However, high-frequency firing can be observed at around P22 [33], coinciding with a significant increase in the expression of Kv3.1 and Kv3.2 between these periods [33,34]. This critical developmental period in mice roughly corresponds to the early stage of human adolescence, also the period when the onset of schizophrenia begins. These observations suggest a potential association between Kv3.1 and Kv3.2 and the development of schizophrenia through impaired function of parvalbumin neurons.

Postmortem brain studies on schizophrenia demonstrated that the impairments in parvalbumin neurons are particularly significant in layer three of the prefrontal cortex, which plays a pivotal role in the formulation of gamma oscillations [35]. Kv3.1 and Kv3.2 expression differs in their distribution, although both are expressed in the cerebral cortex. Kv3.1 is expressed in layers two to six, whereas Kv3.2 is mainly expressed in deep layers five and six in the mouse cortex [36]. Therefore, Kv3.1 is more highly expressed than Kv3.2 in the superficial layers of the cerebral cortex, including layer three. These findings raise the possibility that Kv3.1, rather than Kv3.2, is more potently involved in the functional impairments of parvalbumin neurons in patients with schizophrenia.

Kv3.1 knockout mice exhibit hyperactivity, increased stereotypic behavior, and reduced sleep [37]. In a resting-state EEG, these mice exhibit increased gamma and reduced delta oscillations [38]. The resting-state EEG and behavioral findings in the Kv3.1 knockout mice suggest a state of hyperarousal in these mice. Notably, the onset of patients with schizophrenia typically manifests psychotic symptoms accompanying psychomotor agitation and insomnia. These observations may be associated with behavioral changes in the Kv3.1 knockout mice, characterized by hyperactivity and reduced sleep. Altogether, these lines of evidence suggest the potential involvement of Kv3.1 in the pathophysiology of schizophrenia. Indeed, an animal model study of schizophrenia that used PCP reported a decrease in Kv3.1. In this study, decreased Kv3.1 and parvalbumin mRNA levels were observed in the prefrontal cortex of rats subchronically treated with PCP [39]. This finding strengthens the association between Kv3.1 and the parvalbumin neuronal abnormalities in the pathophysiology of schizophrenia.

### 4.2. Reduced Kv3.1 Expression in the Prefrontal Cortex of Schizophrenia

A postmortem brain analysis of patients with schizophrenia revealed a reduction in Kv3.1 expression, particularly in the prefrontal cortex of untreated patients [40]. In human postmortem brains, the Kv3.1 protein is normally detected in the cerebral cortex but not in subcortical regions, such as the basal ganglia [40]. Contrarily, the Kv3.2 protein was abundantly expressed in both cortical and subcortical regions, but no significant alterations were observed in patients with schizophrenia. In addition, the decrease in cortical Kv3.1 protein levels observed in untreated patients was not present in chronically treated patients with schizophrenia [40]. These findings suggest that Kv3.1 in the cerebral cortex, particularly the prefrontal cortex, plays a pivotal role in the pathophysiology of schizophrenia and may be modulated by antipsychotic medications. These findings may offer a basis for the novel approach to modulate the reduced function of parvalbumin neurons in patients with schizophrenia. This discovery could significantly advance the GABA hypothesis for clinical applications that rescue the dampened firing of parvalbumin neurons through the modulation of the Kv3.1 channel in patients with schizophrenia.

### 4.3. Clinical Trial of the Kv3.1/3.2 Modulator for Patients with Schizophrenia

Since the discovery of reduced Kv3.1 expression in patients with schizophrenia, clinical trials have started exploring Kv3.1/3.2 modulators for the potential treatment of schizophrenia. These drugs are designed to normalize the functional impairments of Kv3.1 and Kv3.2, and in vitro studies have shown promise in restoring high-frequency firing in parvalbumin neurons [41,42]. In recombinant Kv3.1 and Kv3.2 cells, the Kv3.1/3.2 modulator produced a voltage-dependent potentiation of currents, with higher levels of potentiation at voltages close to the threshold for Kv3 channel activation [42]. In mouse brain sections treated with low-concentration tetraethylammonium, a partial blocker of Kv3 channels, the Kv3.1/3.2 modulator, restored the fast-spiking of parvalbumin neurons [42]. Moreover, this modulator had a sedative effect on behavioral hyperactivity in amphetamine and Clock gene mutant mice [43]. Further validation of mechanisms, including the effects of the Kv3.1/3.2 modulator on evoked gamma and other oscillations using animal models, is required. However, these findings indicate that this modulator can be used for treating psychotic disorders. A preliminary clinical experiment using a Kv3.1/3.2 modulator in patients with schizophrenia used positron emission tomography scans to evaluate dopamine synthesis capacity in the striatum [44]. Although the drugs exerted no significant effect on dopamine synthesis, a significant correlation was observed between dopamine synthesis and overall symptom improvement in the group taking Kv3.1/3.2 modulators [44]. In this study, showing a limited impact of Kv3.1/3.2 modulators on dopamine synthesis, there remains a possibility that Kv3.1/3.2 modulators work through mechanisms distinct from directly influencing dopamine, potentially focusing on the improvement of the function of GABAergic neurons.

Another report from the trial of the Kv3.1/3.2 modulator focused on resting-state gamma oscillations as the intermediate phenotype of patients [45]. The patients who initially demonstrated a positive correlation between resting-state gamma power and positive symptoms of schizophrenia exhibited significantly reduced resting-state gamma power just 1-week after drug administration but not at the 4-week mark [45]. Previous studies on patients with schizophrenia have yielded inconsistent results regarding resting-state gamma power, with some studies reporting increases and others showing decreases [46]. However, rodent studies that used NMDA receptor antagonists have demonstrated that these manipulations lead to an increase in resting-state gamma power [46]. Similarly, human studies with NMDA receptor antagonists have reported an increase in resting-state gamma power in healthy individuals [46]. These results are in contrast to the reduction in evoked gamma oscillations typical of the 40 Hz ASSR observed in patients with schizophrenia. The inverse association between resting and evoked gamma oscillations in the schizophrenia model may indicate that parvalbumin neuronal functions are not sufficient to evoke gamma oscillations, at least when baseline gamma oscillations are already elevated because of the disinhibition of principal neurons caused by the dysfunction of parvalbumin and other GABAergic neurons. Altogether, these findings suggest that resting-state gamma power could be a promising biomarker for patient recruitment in future clinical trials of Kv3.1/3.2 modulators.

To date, only one report has compared the clinical effects of the Kv3.1/3.2 modulator with those of a placebo. In this clinical trial, a reduction in positive symptom scores was observed with the Kv3.1/3.2 modulator, although a similar trend was also noted in the placebo [44]. This preliminary finding suggests the need for further studies to determine the clinical efficacy of this modulator. However, it may be important to consider the study design to yield more definitive results. This trial was conducted as add-on therapy to existing antipsychotic medications. Considering the postmortem brain finding that the altered expression of Kv3.1 could be restored with antipsychotic medications [40], it is possible that the medications had already partially restored the Kv3.1 channel function in the patients enrolled in the clinical trial. Therefore, future clinical trials may need to investigate the effects of Kv3.1/3.2 modulatory drugs for patients with schizophrenia who are not under antipsychotic medications. Moreover, further refinement of the target channel may be beneficial. Notably, a novel Kv3.1 positive modulator has been developed [47]. Considering the change in Kv3.1, but not Kv3.2, channels in patients with schizophrenia [40], the potential application of the Kv3.1 positive modulator is anticipated for this population. Such novel medications may offer an alternative approach to managing the symptoms of schizophrenia, including psychosis and, preferably, cognitive impairment, through the restoration of the firing of parvalbumin neurons.

## 5. Utility of 40 Hz ASSR for the Development of Targeted Treatments

### 5.1. Clinical Trials Using 40 Hz ASSR for Patients with Schizophrenia

Robust findings of 40 Hz ASSR impairments in schizophrenia have prompted research into its potential as a biomarker for the diagnosis and prediction of the disease course [48]. While still limited in number, several clinical trials involving patients with schizophrenia have reported 40 Hz ASSR as a possible biomarker for the treatments. In a clinical examination using memantine for patients with schizophrenia, the effect of the drug on the enhancement of the 40 Hz ASSR was reported [49]. In this study, memantine increased the 40 Hz ASSR both in patients with schizophrenia and healthy controls, and the patients administered memantine exhibited an increased 40 Hz ASSR to levels not statistically different from those of healthy controls administered a placebo. However, one study reported that memantine did not significantly enhance the 40 Hz ASSR in healthy controls [29]. These inconsistent results suggest the need for further investigation, taking into consideration factors such as the dosage and duration of the drug trial. NMDA receptors are expressed in GABA and glutamate neurons. Thus, the differential drug effects might be attributed to the various degrees of receptor activation within these neuronal populations, leading to alterations in the inhibition–excitation balance.

**Figure 1:**
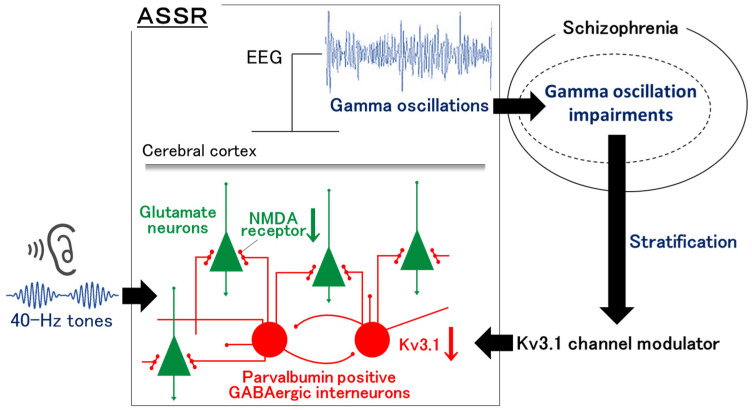
A possible targeted treatment of Kv3 modulator. This figure demonstrates that a Kv3.1 modulator is a promising treatment for schizophrenia in future clinical settings. The auditory steady-state response (ASSR) can help identify patients with significant evoked gamma oscillation impairments. The novel modulator for Kv3.1, the voltage-gated potassium channel that regulates the firing in parvalbumin-positive GABAergic interneurons, may be effective for patients with schizophrenia who were stratified by ASSR impairments. This systematic strategy may pave the way for targeted therapeutics in psychiatry.

Clinical trials of a dopamine D1 receptor modulator and glycine transporter inhibitor have reported results examining 40 Hz ASSR as a biomarker for drug response. The D1 modulator demonstrated a tendency to increase the 40 Hz ASSR, although it was not statistically significant due to the limited sample size [50]. The clinical trial of glycine transporter inhibitor did not report the clear utility of the 40 Hz ASSR as a treatment response biomarker or a predictive biomarker of treatment response [51]. Although these preliminary findings have failed to establish a clear benefit of this approach, a limitation of the study design may be that these studies on the 40 Hz ASSR do not mainly stem from the clinical trials of drugs that have been specifically developed to improve the 40 Hz ASSR. In the future, it is hoped that drugs will be developed based on their improvement effects of the 40 Hz ASSR. This would enable the setting up and design of an alternative therapeutic strategy involving the utilization of the 40 Hz ASSR as a biomarker to stratify patient populations and drug administration (Figure 1). The K3.1 channel modulator may be a potent candidate for this targeted treatment.

### 5.2. Repurposing of an Audizometric Device for Examining 40 Hz ASSR

ASSR is currently employed in otorhinolaryngology for objective hearing assessment with the use of an auditory-evoked response testing device. In this device, EEG electrodes are integrated to record brainwaves in response to the auditory stimuli presented during the test. In otorhinolaryngology, ASSR measurements are typically performed with periodic auditory tones at a frequency of 80–90 Hz during sleep to target brainstem-derived responses, which are less affected by sleep. Contrarily, research on schizophrenia utilizes the 40 Hz ASSR at resting and awake conditions. This approach targets the robust cortical activity induced by the 40 Hz auditory stimulation, allowing for detection in the awake state where background EEG activity is higher than during sleep. However, the 40 Hz ASSR is susceptible to wakefulness levels, highlighting the importance of standardizing the testing condition. The commercially available devices for ASSR used in otorhinolaryngology are optimized for short-time measurements. Such devices can be tuned to emit a 40 Hz auditory stimulus for resting wakefulness testing. Although the parameters obtained in the devices for the ASSR are limited compared with the current methodology used for ASSR research on schizophrenia, the availability of the device was reported for detecting 40 Hz ASSR impairments in patients with schizophrenia [52,53]. Considering their widespread availability globally, these devices could potentially be repurposed with slight modifications for the examination of 40 Hz ASSR impairments for patients with schizophrenia. This approach holds promise for future social implementation in psychiatry due to the existing infrastructure of ASSR devices in clinical settings.

## 6. Potential of Biomarker-Driven Therapeutics in Psychiatry

### 6.1. The Need for Biomarker-Driven Classification for Psychiatric Disorders

Established biomarkers are currently lacking in clinical psychiatry. The clinical assessments for the diagnosis and monitoring of the course of psychiatric disorders heavily rely on clinical interviews, which can be subjective. This lack of objectivity necessitates but also hinders the development of biological interventions for patients with psychiatric disorders from the biological perspective. To overcome these limitations, the Research Domain Criteria project was launched in response to the need to identify objective and dimensional measures of psychiatric disorders based on neurobiological understanding [54].

Accumulating evidence based on these backgrounds highlights the shared biological underpinnings of schizophrenia and related disorders. Large-scale genome- and transcriptome-wide studies have demonstrated shared genetic risks and mechanisms among patients with psychiatric disorders, such as schizophrenia, bipolar disorder, and autism spectrum disorder (ASD) [55,56]. Large-scale brain imaging studies such as BSNP [57,58] and ENIGMA [59,60] consortiums have highlighted the overlapped brain abnormalities between patients with schizophrenia and those with bipolar disorder. In addition, a genome-wide study reported that the common symptoms observed in both patients, such as psychosis, were associated with shared genetic risks [61]. In clinical practice, antipsychotics are often prescribed not only for schizophrenia but also for other disorders with psychotic symptoms, which may reflect the shared biological underpinnings. These lines of evidence highlight the need for biomarker-driven therapeutics that are not restricted by current diagnostic labels for psychiatric disorders.

Abnormalities in parvalbumin neurons and impairments in evoked gamma oscillations are not specific to patients with schizophrenia. Postmortem brain studies have demonstrated that abnormalities in parvalbumin neurons were detected in patients with bipolar disorder and ASD [62]. Similarly, impairments in evoked gamma oscillations were observed in patients with bipolar disorder [63] and ASD [64]. The GABA hypothesis may provide a basis for the development of biomarker-driven therapeutics that target parvalbumin neuronal transmissions across a broad spectrum of schizophrenia-related disorders.

### 6.2. A Paradigm Shift in Clinical Psychiatry through Biomarker-Driven Therapeutics

The development of targeted treatments has the potential to innovate therapeutic strategies for schizophrenia and related disorders. In the current clinical system, patients are evaluated and diagnosed based on clinical interviews, a process that remains invisible to them. The introduction of biomarkers can provide patients and physicians with clear information about detectable biological abnormalities. This transparency may be transformative, particularly for the treatment of schizophrenia. Delusion, a core symptom of schizophrenia and a target of antipsychotic medications, exemplifies this challenge. Patients with schizophrenia often experience a significant disconnection between their own perception of reality and that of people around them, which typically presents as delusion, i.e., fixed and false beliefs. It is difficult to share delusion itself as the therapeutic target between patients and physicians as it is the perception believed by the patient but is discrepant from what other people, including the attending physician, experience. Such a discrepancy may make it difficult to find common ground for treatment goals between the patient and the attending physician. Biomarker-driven therapeutics provide a potential solution. By shifting the focus from the discrepant perception to detectable biomarkers, the sharing of the treatment goals could be promoted, which may improve communication and collaboration between the patient and physician. This shift has the potential to fundamentally modify how we approach schizophrenia and related disorders. It may lead to more effective outcomes for patients with these disorders by moving away from a psychopathological perspective, sometimes believed to be a lack of insight into their illness.

## 7. Conclusions

Schizophrenia is a complex disorder with a heterogeneous pathophysiology. Future pharmacological treatments may shift toward biomarker-driven stratification and therapeutics for patients with schizophrenia and related disorders. In this context, the GABA hypothesis of schizophrenia offers a promising avenue for the development of novel biomarker-driven therapeutics by targeting the dysfunction of parvalbumin neurons. This targeted approach has the potential to move from dopamine receptor antagonism and pave the way for more personalized, patient-centered treatments based on objectively visible biomarkers. Ultimately, such advancements could provide a more refined understanding of schizophrenia and related disorders and potentially facilitate the future development of strategies for psychiatric diagnoses and their treatments.

## Data Availability

No new data were created or analyzed in this study.
